# Tegafur Substitution for 5-Fu in Combination with Actinomycin D to Treat Gestational Trophoblastic Neoplasm

**DOI:** 10.1371/journal.pone.0143531

**Published:** 2015-11-23

**Authors:** Mei Peng, Yiling Ding, Ling Yu, Yali Deng, Weisi Lai, Yun Hu, Hongwen Zhang, Xianqing Wu, Hong Fan, Hui Ding, Yilin Wu, Guangshi Tao

**Affiliations:** Department of Gynaecology & Obstetrics, the Second Xiangya Hospital of Central South University, Changsha 410011, China; Zhejiang University School of Medicine, CHINA

## Abstract

Although 5-fluorouracil (5-Fu) combination chemotherapy provides a satisfactory therapeutic response in patients with gestational trophoblastic neoplasms (GTNs), it has severe side effects. The current study analyzed the therapeutic effects and side effects of tegafur plus actinomycin D (Act-D) vs. 5-Fu plus Act-D for the treatment of GTNs based on controlled historical records. A total of 427 GTN cases that received tegafur and Act-D combination chemotherapy at the Second Xiangya Hospital of XiangYa Medical School between August 2003 and July 2013 were analyzed based on historical data. A total of 393 GTN cases that received 5-Fu plus Act-D between August 1993 and July 2003 at the same hospital were also analyzed, which constituted the control group. The therapeutic effects, toxicity and side effects after chemotherapy were compared between the groups. The overall response rate was 90.63% in the tegafur+Act-D group (tegafur group) and 92.37% in the 5-Fu+Act-D group (5-Fu group); these rates were not significantly different (P > 0.05). However, the incidence rates of myelosuppression (white blood cell decline), gastrointestinal reactions (nausea, vomiting, dental ulcer, and diarrhea), skin lesions and phlebitis were lower in the tegafur group than in the 5-Fu group (P < 0.05). The results of this study may provide useful data for the clinical application of tegafur in GTN treatment.

## Introduction

Gestational trophoblastic neoplasms (GTNs) are a group of highly malignant carcinomas, including invasive hydatidiform mole (IHM), choriocarcinoma (CC) and placental site trophoblastic tumor (PSTT). Because of its particularity, studies on GTNs usually exclude PSTTs. Approximately 60% of GTNs occur secondary to a hydatidiform mole, 30% occur after abortion, and 10% appear after full-term pregnancy or ectopic gestation [1]. The GTN morbidity varies, with greater morbidity in Asia than in Europe or the USA. GTN morbidity is influenced by the natural population and the patient’s medical condition and educational background. Studies in the last 10 years have indicated that the morbidity associated with GTNs has been declining [2, 3]. Before the development of efficient chemotherapeutics, surgery was the major treatment strategy, with a high mortality rate of 80%-90% [4, 5]. In the 1950s, efficient chemotherapeutics were developed, including single agent methotrexate (MTX), single agent 5-Fu and the combination of 5-Fu and Act-D. Since, these chemotherapeutic regimens have been used extensively worldwide [6, 7, 8]. The cure rate after chemotherapy alone is over 90% in non-metastatic cases and averages 80%-90% overall [9]. The high sensitivity to chemotherapy and the high sensitivity and high specificity of β-HCG neoplasia monitoring have enabled GTNs to be associated with the best prognosis among all types of malignancies [10, 11, 12].

The curative effects of 5-Fu monotherapy and combination therapy with 5-Fu and Act-D have been reported in GTN [13–15]. However, in some cases, 5-Fu therapy causes side effects (e.g., severe gastrointestinal reactions and marrow suppression) that often result in suspended, postponed, or cancelled treatment, which indicates treatment failure [16]. Tegafur, a derivative of 5-Fu, was synthesized by Hiller in 1966 and evaluated in a clinical trial in 1968. The anti-tumor spectrum of tegafur is similar to that of 5-Fu, and it is used mainly in digestive tract neoplasms [17]. Tegafur is activated in the liver and is gradually converted into fluorouracil, which can inhibit or block the synthesis of DNA, RNA and proteins and suppress tumor development. Tegafur, a cell cycle-specific drug, functions mainly in S phase and has a similar mechanism of action, curative effect and anti-tumor spectrum as fluorouracil. Moreover, tegafur has the advantages of sustained activity, lower toxicity, and a higher chemotherapeutic index. The anti-tumor activity of tegafur has been broadly demonstrated in clinical studies for the treatment of gastrointestinal tumors, such as gastric carcinoma, rectal carcinoma, pancreatic carcinoma and liver cancer, and utilized in chemotherapy regimens for other malignancies, such as breast cancer, lung cancer and nasopharyngeal cancer. Based on its therapeutic effects and minimal side effects, tegafur has been used extensively worldwide [17–21]. However, to the best of our knowledge, the use of tegafur for GTN treatment has not yet been reported.

Based on the aforementioned data, this study compared the therapeutic efficacy and toxicity profile of tegafur plus Act-D vs. 5-Fu plus Act-D in the treatment of GTN based on controlled historical records. The results may provide evidence for the increased clinical application of tegafur for GTN treatment.

## Methods

### General data

A total of 427 consecutive GTN cases that received tegafur+Act-D combination chemotherapy between August 2003 and July 2013 at the Second Xiangya Hospital of XiangYa Medical School were analyzed based on historical records. These cases constituted the study group. To investigate the therapeutic effects, toxicity and side effects of tegafur+Act-D, 393 consecutive GTN cases that received 5-Fu+Act-D at the same institute between August 1993 and July 2003 were also analyzed based on historical data. These cases constituted the control group.

All the cases were analyzed from the time of initial treatment. The inclusion criteria for patients with a post-hydatidiform mole GTN were based on the diagnostic standard authorized in 2000 and issued in 2004 [22], and the criteria for a diagnosis of post-non-hydatidiform mole GTN were based on the diagnostic standard proposed by Peking Union Medical College Hospital [8]. Staging criteria were based on the new clinical staging standard authorized in 2000 and issued in 2002 by the FIGO Committee of Gynecological Oncology [23].

All the cases that met the diagnostic criteria were included. Disease staging and scoring were performed for all cases based on the anatomic staging and prognosis scoring system, FIGO 2000. A prognosis score of ≤ 6 was assessed as low-risk, and that of ≥7 was assessed as high-risk.

This study was approved by the ethics committee of the Second Xiangya Hospital of Central South University. The need to obtain informed consent was waived by the committee.

### Treatment methods

The combination treatment of tegafur and Act-D was evaluated in this study. The daily dose of tegafur was 26–27 mg/kg [24] administered intravenously with 500 mL of 5% glucose within 3–4 hours. The daily dose of Act-D was 6–7 μg/kg administered intravenously with 500 mL of 0.9% NaCl within 3–4 hours. In the 5-Fu and Act-D combination treatment group, the daily dose of 5-Fu was 26–27 mg/kg in 500 mL of 5% glucose administered within 6–8 hours, and Act-D was administered as described above. Each treatment course was 8 days long, and the course interval was 3 weeks. The resolution of symptoms, signs, the primary lesion, and metastasis were considered indications for drug discontinuance. The treatment was consolidated to 1–2 courses for low-risk patients or 2–3 courses for high-risk patients after β-HCG levels decreased to the normal range.

### Evaluation of therapeutic effects and side effects

This evaluation was based on the curative effect standards in gestational trophoblastic disease treatment and the grading standards for acute and subacute toxic reactions to anti-tumor drugs proposed by the WHO.

Peripheral hemograms were examined on alternate days, and hepatic and renal function was examined weekly. A complete evaluation was conducted once during each course of treatment, 21 days after the treatment course and prior to the next course. This evaluation included β-HCG measurements and gynecologic, ultrasound, CT and lung X-ray examinations. The evaluation standard includes complete response (CR), partial response (PR), stable disease (SD) and progressive disease (PD). Normal β-HCG levels in 3 consecutive weeks indicate a CR, which entails the disappearance of clinical signs and metastasis (based on pelvic cavity ultrasound, lung X-ray, and lung and liver CT). In cases of SD, the β-HCG level declines or increases no more than 10% with no significant change in clinical signs, and the measurable lesion shrinks less than 50% or grows by no more than 25%. In PD, β-HCG levels increase, and clinical signs become more severe. In addition, the measurable lesion increases by more than 25%, or a new lesion emerges [25, 26].

The clinical criteria for a cure or drug discontinuance include the disappearance of clinical symptoms and signs and 3 consecutive normal weekly β-HCG measurements followed by a consolidated 2–3 courses of chemotherapy. The drug resistance criteria include no significant decline in β-HCG after 2 therapeutic courses (not exceeding 1/10), stable β-HCG levels or an increasing trend in β-HCG levels. Based on other examinations, the lack of an increase or decrease in lesion size or the development of a new lesion indicates that the treatment had no effect; converse data indicate effective treatment [22].

### Statistical analysis

Data are presented as the mean ± standard error ($\bar{x}$ ± s), and the data were analyzed using SPSS 17.0. The *X* ^*2*^ text was used to compare enumerated data in the two groups. Continuous and categorical variables were analyzed with a t test. P < 0.05 was considered to be statistically significant.

## Results

The general data on the two groups are summarized in Table 1. The type of previous pregnancy, pre-treatment β-HCG levels, maximum tumor diameter, and the total number of treatment courses required for β-HCG to return to normal are summarized in Table 2. No significance differences in these data were observed between the two groups (P < 0.05).

**Table 1:** General data.

Data	5-Fu	Tegafur
Case (n)	393	427
Age (years)	30.68±4.42	30.79±4.41
Disease type (n)		
	Invasive Hydatidiform Mole (273)	Invasive Hydatidiform Mole (325)
	Chorioepithelioma (120)	Chorioepithelioma (102)
Clinical stage (n)		
	I (244)	I (235)
	II (96)	II (108)
	III (52)	III (82)
	IV (1)	IV (2)
Prognosis (n)		
	Low risk (271)	Low risk (297)
	High risk (122)	High risk (130)

**Table 2:** Type of previous pregnancy, pre-treatment β-HCG levels, maximum tumor diameter, and the total number of treatment courses to achieve normal β-HCG levels ($\bar{x}$ ± s).

Index	5-Fu	Tegafur
Type of previous pregnancy (n)		
	Hydatidiform mole (311)	Hydatidiform mole (345)
	Abortion (52)	Abortion (50)
	Term birth (30)	Term birth (32)
Log-value of pre-treatment β-HCG	4.04±0.79	3.99±0.82
Maximum tumor diameter (cm)	3.67±2.43	3.75±2.27
Total number of treatment courses to achieve normal β-HCG levels	3.97±1.45	4.00±1.16

The complete response rates in the tegafur and 5-Fu groups were 74.24% and 76.59%, respectively, and the overall response rates were 90.63% and 92.37%, respectively. There were no significant differences in the therapeutic efficacy between these two groups (Table 3, P > 0.05), and neither the complete response rate nor the overall response rate was significantly different (P > 0.05).

**Table 3:** Comparison of therapeutic effect between the tegafur and 5-Fu groups.

Index	5-Fu	Tegafur
Total	393	427
CR	301	317
PR	62	70
SD	19	26
PD	11	14
CR+PR	363	387
Overall response rate (%)	92.36	90.63
*P*	>0.05	

CR, complete response. PR, partial response. SD, stable disease. PD, progressive disease.

The chemotherapeutic side effects in the two groups were compared, and the results are shown in Table 4. The incidence of myelosuppression with a decrease in white blood cell (WBC) count was 62.76% (268/427) in the tegafur treatment group and 82.19% (323/393) in the 5-Fu group; the difference between the two groups was significant (P = 0.004). There was one death (0.25%, 1/393) as a result of severe myelosuppression in the 5-Fu group. In the tegafur group, the incidence rates of nausea and vomiting, dental ulcer, and diarrhea were 72.13% (308/427), 17.33% (74/427) and 8.90% (38/427), respectively.

**Table 4:** Comparison of the side effects between the tegafur and 5-Fu groups.

Group	Number of cases	WBC decrease				Nausea and vomiting				Dental ulcer				Diarrhea				Skin lesion			
		I	II	III	IV	I	II	III	IV	I	II	III	IV	I	II	III	IV	I	II	III	IV
5-Fu	393	159	97	58	9	99	125	165	4	76	95	116	3	40	79	48	2	41	72	47	1
Tegafur	427	155	87	26	0	60	67	31	0	55	19	0	0	22	16	0	0	7	0	0	0
*P*	0.004					*< 0*.*001*				*<0*.*001*				*< 0*.*001*				*< 0*.*001*			

In the 5-Fu group, the incidence rates of nausea and vomiting, dental ulcers, and diarrhea were 88.04% (346/393), 73.79% (290/393), and 43.00% (169/393), respectively. In addition, there was one 0.25% (1/393) death in the 5-Fu group. Significant differences were observed in the incidence of gastrointestinal side effects between the groups (P < 0.001). In the tegafur group, 7 cases (1.64%, 7/427) of grade I skin lesions were observed, but there were no grade II, III or IV skin lesions. In the 5-Fu group, the overall incidence of skin lesions was 41.98% (165/393), with 10.43% (41/393) grade I, 18.32% (72/393) grade II, 11.96% (47/393) grade III and 0.03% (1/393) grade IV. The differences between the two groups were statistically significant (P < 0.001).

Hepatic dysfunction was observed in 29.27% (125/427) of the patients in the tegafur group and 35.37% (139/393) of those in the 5-Fu group. The difference between the two groups was not statistically significant (P > 0.05). The phlebitis incidence was 3.04% (13/427) in the tegafur group and 54.20% (213/393) in the 5-Fu group; this difference was statistically significant (P < 0.05). The incidence rates of drug resistance, alopecia, fever and neural system changes in the tegafur group and 5-Fu group were not statistically significant (P > 0.05). The average inpatient stay was shorter and the average hospitalization cost was less in the tegafur group compared with the 5-Fu group, but these differences were not statistically significant (P > 0.05).

In the tegafur group, 411 patients returned for follow-up visits, and 16 patients were lost. Among these 411 patients, 211 were followed for 5 years with a relapse rate of 0.48% (2/411), and 163 were followed for 2–5 years with a relapse rate of 0.24% (1/411). The remaining 37 patients were followed for more than 1.5 years, and no relapses occurred. In the 5-Fu group, 378 patients completed a follow-up visit, and 15 patients were lost. Among these 378 patients, the recurrence rate at the 5-year visit was 0.79% (3/378). The differences between the two groups were not statistically significant (P > 0.05).

## Discussion

GTNs have been classified into a group of highly malignant tumors. Treatment strategies, such as the administration of single agent MTX, single agent 5-Fu or combination 5-Fu+Act-D therapy, have been broadly accepted [6, 7, 8, 26]. A single drug multicourse strategy is used primarily in chemotherapy for low-risk trophoblastic neoplasms, but this regimen has a 10%-30% failure rate as preliminary chemotherapy. This situation could significantly lengthen the treatment duration and postpone pregnancy. Therefore, it was determined that combination chemotherapy could be utilized to treat low-risk trophoblastic neoplasms to obtain higher remission rates after preliminary chemotherapy, to shorten the treatment time and to decrease toxicity and side effects. In some cases, even with satisfactory therapeutic effects, combination chemotherapy can cause severe myelosuppression and digestive tract symptoms, resulting in suspension of chemotherapy [27]. The major component of tegafur is a latent effective derivative that gradually releases 5-Fu through the removal of tetrahydrofuran by the P450 enzyme in the liver. Inhibition of deoxythymidine by fluorodeoxyuridine, the active metabolic product of 5-Fu, interferes with the conversion of deoxyuridine to deoxythymidine. This process can block the synthesis of DNA, RNA and proteins in a cell cycle-specific pattern and elicit an anti-tumor effect [28, 29]. Tegafur, an anti-pyrimidine drug with a cell cycle-specific pattern of activity, functions in S phase and has a similar mechanism of action, therapeutic effect and anti-tumor spectrum as fluorouracil but a lower toxicity profile. After intravenous administration and a relatively mild metabolic process, tegafur exhibits moderate activity, and higher blood concentrations of tegafur compared with 5-Fu are sustained over 12 hours with stable efficacy [30]. Animal experiments demonstrated that the toxicity of tegafur is only 1/4-1/7 that of 5-Fu, and the chemotherapeutic index is 2-fold that of 5-Fu [16]. No severe myelosuppression was observed in experiments on the chronic toxicity of tegafur, indicating that this drug has only a slight effect on immunity [29, 31, 32].

5-Fu plays an important role in GTN chemotherapy. The most commonly utilized chemotherapies for GTN treatment are single agent 5-Fu and 5-Fu combination chemotherapy, including 5-Fu+Act-D or 5-Fu+EMA-CO. In western countries, 5-Fu+EMA-CO therapy is the primary approach. However, a prolonged hospitalization is often required due to the short interval (one week) between the two treatment courses, which results in noncompliance. In China, the most commonly used therapy is 5-Fu+Act-D, which has an interval of three weeks between treatment courses. Tegafur has been reported to possess broad anti-tumor activity in clinical studies [28], but its application in GTN chemotherapy has not been reported. The response rate to tegafur therapy in combination with other anti-tumor drugs in gastrointestinal tumors is approximately 50%-70%. Ajnai et al. reported that the response rate to the combination of tegafur and Nedaplatin was 51% in patients with esophageal cancer [33], and Li et al. reported that the response rate to this treatment regimen was 56.8% in patients with advanced esophageal cancer in China [34]. Other scholars have reported response rates to tegafur/Nedaplatin combination treatment of greater than 70% in patients with advanced esophageal cancer [35]. However, the response rates reported in the literature are all lower than what is reported in the present study, potentially because 5-Fu is a very effective chemotherapeutic agent in GTN treatment; trophoblastic neoplasms themselves are highly malignant tumors with the highest cure rate among all tumor types. The response rate to 5-Fu in patients with GTNs outside China is often approximately 90%; meanwhile, the response rates to 5-Fu in patients with other types of cancer are significantly lower. Our results showed that the response rates to tegafur and to 5-Fu were similar in patients with trophoblastic tumors. Another reason for the high cure rate in this study is the combination chemotherapy approach; a single chemotherapeutic drug cannot achieve a 100% cure rate in low-risk patients, but the cure rate among low-risk patients in the present study was 100%. Therefore, the overall cure rate was slightly higher than what has been reported in the literature.

The chemotherapeutic side effects observed in the 5-Fu group were mainly myelosuppression and digestive tract symptoms. Myelosuppression primarily manifested as a decrease in WBC count, and the digestive tract symptoms included nausea and vomiting, dental ulcers, and diarrhea. In severe cases, the symptoms manifested as depression, loss of appetite, and pseudomembranous colitis induced by water and electrolyte imbalances, which can lead to death if not properly treated. Some patients had to interrupt or delay chemotherapy, and certain patients ceased treatment due to unbearable and serious chemotherapeutic side effects, leading to the failure of chemotherapy. In addition, because the anti-tumor activity of 5-Fu is time-dependent, continuous central venous catheters and intravenous infusion are required, but they can cause inflammation, infection and blood clots within veins, thereby increasing treatment cost and lengthening hospital stays. Moreover, severe phlebitis and skin lesions developed in a few patients, significantly affecting the quality of life during chemotherapy; therefore, most patients are unable to tolerate multi-course chemotherapy. The metabolism of tegafur into 5-Fu is an enzyme-mediated process that involves the P450 enzyme in the liver and thymidine phosphorylase (TP) in tumor tissue. Because the conversion of tegafur to 5-Fu requires the participation of TP, which is at a much higher concentration in solid tumors than in normal tissues, 5-Fu is preferentially delivered to tumor tissues [28]. The preferential distribution of 5-Fu in tumor tissue is a favorable factor for clinical treatment because the tumor-specific increased concentration of this cytotoxic compound enhances the anti-tumor activity, while the lower 5-Fu plasma concentration reduces the incidence of serious systemic adverse drug reactions. The results from this study showed that the incidence rates of decreased WBC count, gastrointestinal symptoms, and other side effects such as skin lesions and phlebitis during treatment were significantly lower in the tegafur group than in the 5-Fu group.

This study has some limitations. First, it is a controlled historical data analysis; therefore, the clinical treatment, laboratory examinations, and prevention and treatment of toxicity and side effects in all patients may not have been the same as what would be observed in controlled studies under the same conditions. Second, this study is restricted by the diagnostic and treatment options that were available during the analyzed time period, such as the auxiliary examination equipment, the sensitivity of reagents in detecting indicators, the prevention and treatment of side effects during chemotherapy, general patient nutrition, and the level of education, which may have influenced patient outcome. More accurate clinical information should be obtained in future randomized controlled studies. Third, fewer patients in the tegafur group compared with the 5-Fu group completed the 5-year follow-up visit, which may have influenced the analytical results of this study.

The combination of tegafur with Act-D appeared to be a more effective treatment for GTNs than the combination of 5-Fu and Act-D. Compared with the 5-Fu group, the main advantages of tegafur are better patient tolerance, milder toxicity and side effects, less severe myelosuppression, fewer gastrointestinal reactions, and less severe phlebitis and skin lesions. Although similar outcomes were achieved, no lengthy intravenous infusions of chemotherapeutic drugs were required, which greatly improved the quality of life of the patients, particularly that of infirm patients, whose tolerance is poor. This therapy is a potential new choice for the treatment of GTNs that warrants further research and promotion in clinical practice.
